# Digital PCR methods improve detection sensitivity and measurement precision of low abundance mtDNA deletions

**DOI:** 10.1038/srep25186

**Published:** 2016-04-28

**Authors:** Frances R. Belmonte, James L. Martin, Kristin Frescura, Joana Damas, Filipe Pereira, Mark A. Tarnopolsky, Brett A. Kaufman

**Affiliations:** 1 University of Pittsburgh School of Medicine, Division of Cardiology, Center for Metabolism and Mitochondrial Medicine and Vascular Medicine Institute, Pittsburgh, PA, 15261, United States; 2 Departments of Pediatrics and Medicine, McMaster University Medical Center, Hamilton, ON, L8N 3Z5, Canada; 3 Department of Comparative Biomedical Sciences, Royal Veterinary College, London, UK; 4 Interdisciplinary Centre of Marine and Environmental Research (CIIMAR), University of Porto, Porto, Portugal

## Abstract

Mitochondrial DNA (mtDNA) mutations are a common cause of primary mitochondrial disorders, and have also been implicated in a broad collection of conditions, including aging, neurodegeneration, and cancer. Prevalent among these pathogenic variants are mtDNA deletions, which show a strong bias for the loss of sequence in the major arc between, but not including, the heavy and light strand origins of replication. Because individual mtDNA deletions can accumulate focally, occur with multiple mixed breakpoints, and in the presence of normal mtDNA sequences, methods that detect broad-spectrum mutations with enhanced sensitivity and limited costs have both research and clinical applications. In this study, we evaluated semi-quantitative and digital PCR-based methods of mtDNA deletion detection using double-stranded reference templates or biological samples. Our aim was to describe key experimental assay parameters that will enable the analysis of low levels or small differences in mtDNA deletion load during disease progression, with limited false-positive detection. We determined that the digital PCR method significantly improved mtDNA deletion detection sensitivity through absolute quantitation, improved precision and reduced assay standard error.

The human mitochondrial genome (mtDNA) is a multicopy, circular double-stranded molecule residing in the mitochondrial matrix. Human mtDNA is a gene-dense, intronless 16,569 kb genome encoding thirteen essential protein components of the mitochondrial respiratory chain and 24 RNAs required for their expression (Fig. 1). Respiratory dysfunction related to the mitochondrial genome can be subcategorized into mtDNA depletion (low copy number), point mutations, and deletions (reviewed in Schon *et al.*^1^). Clinical cases of depletion are rare, possibly due to mRNA compensatory mechanisms^2^,^3^. As a significant contributor to mitochondrial disease, and the focus of this study, mtDNA deletions are attributed to at least 20% of all mitochondrial disease cases^4^.

The positions of mtDNA deletions are non-random^5^,^6^,^7^. A map of all mtDNA deletion breakpoints reported in MitoBreak^8^, a database of unique mtDNA deletions, is shown in the Circos plot in Fig. 1. Of these mtDNA deletions, loss of the ND4 sequence occurs in approximately 90% of all reported breakpoints^9^,^10^. Most likely this is an underestimation of representative ND4 loss in the patient population, as the frequency of a single deletion was not represented. One example is the so called “common” deletion, which lacks 4977 bp, inclusive of the ND4 sequence^11^. This common deletion causes primary mitochondrial diseases, including Kearns-Sayre syndrome (KSS), Pearson’s syndrome, and chronic progressive external ophthalmoplegia (CPEO)^4^,^12^,^13^,^14^. Of emerging interest is its association with age-related diseases susceptibility, such as cancer, muscle atrophy and neurodegeneration^15^,^16^,^17^.

In both inherited and acquired mtDNA deletions, affected tissues have focal accumulation of mtDNA deletions^18^, but are in the continued presence of varying amounts of normal mtDNA, a condition termed heteroplasmy. Within a tissue, a small but pathophysiologically significant proportion of cells contain a high percentage of mtDNA deletions, yet the total mtDNA deletion burden in the whole tissue is low, posing a challenge for their quantitation. Thus, mtDNA deletions are frequently measured by performing tissue biopsy, followed by histochemical staining for respiratory activity (e.g. cytochrome *c* oxidase and succinate dehydrogenase activities^19^), and then laser-capture microdissection of dysfunctional regions to enrich for pathogenic mtDNA variants^20^,^21^,^22^. Isolated DNA is then analyzed by a variety of mtDNA deletion detection methods, albeit with limitations. For example, Southern blotting requires large amounts of DNA and is limited to single rather than multiple deletions^23^,^24^. Long-range PCR is used to amplify smaller (i.e. deleted) genomes, but requires high-quality intact mtDNA, can be affected by amplification bias toward smaller PCR products^25^,^26^, and is prone to PCR-mediated artifacts such as deletions formed during amplification^27^. Although next-generation sequencing (NGS) is improving in its application for mtDNA deletion detection^28^, it requires deep sequence coverage, which for experimental application is expensive, time consuming and bioinformatics intensive. Furthermore, most NGS techniques utilize amplification prior to library generation, warranting concern about amplification bias causing over estimation^29^. For cost and sensitivity reasons, relative quantitative PCR (qPCR) methodologies are frequently used for qualitative mtDNA deletion burden assessment.

Quantitative PCR has several benefits when compared to the above approaches. It can be used on reasonably small amounts of each sample compared to Southern blot approaches. The short amplicons used in qPCR do not show the amplification bias observed in long template samples and can be paired for additional analyses such as relative mtDNA content. Furthermore, this approach can be successfully used to detect both single and multiple mtDNA deletions. As with all of the methods mentioned above, the most significant challenge is quantitative detection of low abundance mtDNA deletions from human samples without histochemical testing and enrichment.

Single molecule PCR (smPCR) methods have been developed to increase the sensitivity for measuring mtDNA deletions. In smPCR, DNA templates are diluted such that a majority of amplification compartments (i.e. well or droplet) receive 0 or 1 DNA molecule prior to amplification. In contrast to qPCR, which assigns a quantitation cycle value (C_q_) to each well based on a fluorescence detection threshold during amplification, smPCR is an end-point analysis whereby the amplified products are resolved on agarose gels and scored for the presence of shortened amplification products resulting from mtDNA deletions. The smPCR method has been applied to mtDNA deletions using both short^30^ or long amplicons^31^. This approach requires the development of effective PCR primers, and the number of PCR-positive gel lanes analyzed limits the precision of this technique.

More recently, digital PCR (dPCR) methods have been employed to allow the detection of mtDNA deletions^32^. Digital PCR is a refinement of the smPCR, as it partitions thousands to millions of reaction compartments to increase precision but like qPCR uses fluorescence detection rather than gel analysis. The enhanced sensitivity from dPCR permits the detection of rare events, such as detection of low-level pathogens, or precise copy number variation without the use of external standards. Although digital approaches have been used to quantify mtDNA deletions, this effort has been limited to high deletion abundance^32^. Low abundance mtDNA deletions have been detected by combining restriction enzymes^33^ and either qPCR or next generation sequencing^32^,^33^,^34^,^35^.

In the current study, we describe the detection limits for the majority of mtDNA deletions using qPCR, which are improved significantly by quantitation with dPCR. Furthermore, dPCR determines the absolute copies of DNA sequence without need for an external reference that lacks mtDNA deletions, allowing direct assessment of any sample. To our knowledge, this is the first time that both methodologies have been compared to show the advantages of dPCR in directly quantifying low abundance mtDNA deletions over qPCR-based methods that utilize external calibration.

## Results

### Relative mitochondrial DNA content assays

**Figure 2:**
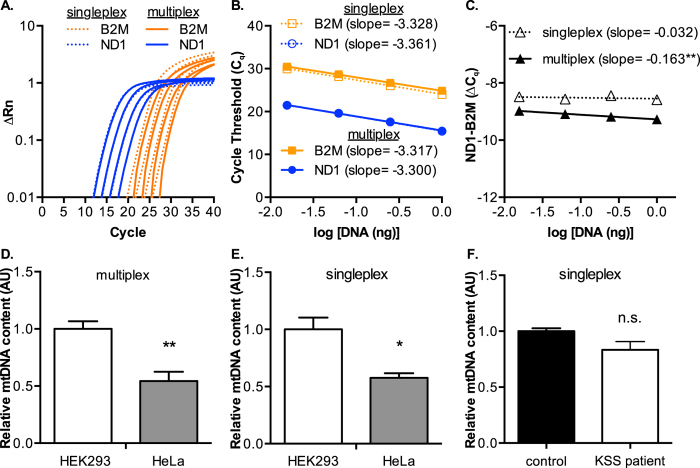
Comparison of multiplex and singleplex mtDNA copy number assays using TAQMAN PCR probes. (**A**) Log plot of fluorescence signal normalized to baseline (∆Rn) for ND1 (Hex) and B2M (FAM) TAQMAN probes in a dilution series of HeLa total DNA preparation comparing singleplex to multiplex assays. Triplicate replicates at each dilution were performed and shown in the figure is a single, representative curve for each dilution. (**B**) Median C_q_ values plotted as a function of total DNA analyzed in singleplex and multiplex assays Three DNA isolate samples from each cell line were used per experiment. Slopes were determined by linear regression (all r^2^ ≥ 0.99). (**C**) Delta C_q_ values plotted as a function of DNA concentration. (**D**) Relative mtDNA content of HeLa and HEK cell lines in a multiplex and (**E**) singleplex assays (significance determined by t-test). (**F**) Relative mtDNA content of control human fibroblast and “common deletion” patient fibroblast cell lines determined by ND1 and B2M singleplex assays. Experiments were performed with two DNA isolate samples per cell line and were assayed in technical triplicates. Significance p-values were: * < 0.05; ** < 0.01.

Assessment of mtDNA levels prior to mtDNA deletion analysis is a common first step in sample characterization, which also provides C_q_ values used for establishing DNA dilutions in later analyses. For our studies, relative mtDNA abundance between samples was measured using the mitochondrial ND1 and nuclear B2M TAQMAN probes previously described^24^. To optimize our approach, we first addressed the potential differences in singleplex or multiplex reactions using ND1 (primer limited) and B2M on total HeLa DNA (Fig. 2). Representative curves for each dilution of total HeLa DNA show that multiplex reactions cause a shift in the fluorescent signal curves (ΔRn) from B2M, but not ND1, and is likely a result of some reagent depletion prior to B2M amplification (Fig. 2A). This shift is non-consequential, as linear regression analyses calculated from the median C_q_ values as a function of total DNA show that the slopes of ND1 and B2M in singleplex and multiplex assays are in close agreement (Fig. 2B). When the delta C_q_ (∆C_q_) values were plotted against DNA concentration, the slope of the multiplexed reaction (−0.163) revealed a modest effect of DNA concentration on the ∆C_q_ values (Fig. 2C).

To validate the multiplex and singleplex assays for relative mtDNA abundance, we prepared total DNA from the HEK293 cell line, which is known to have a higher abundance of mtDNA in comparison to the HeLa cell line^36^. Our data show that the relative mtDNA content in HEK293 cells is approximately double that of HeLa cells, in both multiplex (Fig. 2D) and singleplex (Fig. 2E) when using triplicate DNA isolates from each cell line. We find that multiplex reactions generally show reduced error, thus increasing the statistical significance (p-values) for differences between samples (data not shown). Results from a singleplex reaction with control and “common deletion” KSS patient fibroblast cell lines that were assayed in triplicate at two dilutions do not show any differences in relative mtDNA content as determined by the median C_q_ values of ND1 and B2M (Fig. 2F).

### Validation of a multiplexed ND1 and ND4 TAQMAN assay using a ND1 to ND4 mtDNA PCR product

**Figure 3:**
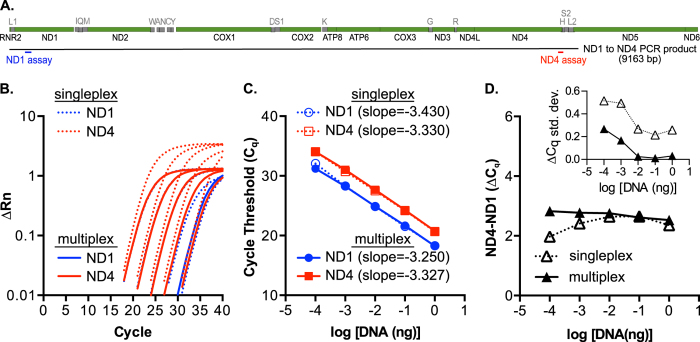
Qualifying ND4 and ND1 TAQMAN assays on a model mtDNA unimolecular substrate. (**A**) Linear diagram showing the eluted PCR product ranging from ND1 through ND4 (9163 bp) used as a reference, deletion-free, double-stranded substrate for qPCR assays. Location of ND1 and ND4 assays are indicated in blue and red, respectively. (**B**) Log plots of the ∆Rn traces for singleplex and multiplex ND1 and ND4 assays on a dilution series of ND1 to ND4 PCR product. A single, representative curve for each dilution is shown; triplicate replicates were performed for each dilution. (**C**) Average C_q_ values plotted for the log of substrate (ng). Slopes were determined by linear regression analysis (all r^2^ ≥ 0.99). (**D**) ND4 minus ND1 (ΔC_q_) values as a log function of DNA template concentration in singleplex and multiplex reactions. Inset: Standard deviation as a function of log of substrate shows lower variability in the multiplex reaction and with higher concentrations of DNA.

Prior to testing mtDNA deletion burden in patient samples, we first characterized the ND1 and ND4 TAQMAN assays on a model template (Fig. 3). To that end, we generated and purified a PCR product containing intact ND1 and ND4 sequence (ND1^+^ND4^+^) to serve as a reference no-deletion mtDNA substrate, illustrated in Fig. 3A. Using this template in both singleplex and multiplex reactions with ND1 and ND4 assays, we found a shift in the ND4 baseline of the ∆Rn plots in singleplex reactions (Fig. 3B) and modest effects on linear regression analysis for C_q_ values throughout the template concentrations tested (Fig. 3C). The ∆C_q_ values across the tested range were highly inconsistent in singleplex reactions (Fig. 3D), particularly with lower concentrations of DNA as shown by the curved line in the singleplex reaction compared to the improved linearity for the multiplex reaction. Higher ∆C_q_ variability is associated with diluted samples that contain less DNA (Fig. 3D, inset). These data show a specific benefit of the multiplex reactions with DNA dilutions that yield ND1 C_q_ values less than 25 in this experimental system. Thus, by using the ND1-to-ND4 non-deletion PCR product as a mtDNA model substrate, we are able to define the qPCR parameters for detecting ND1 and ND4, which are used in subsequent mtDNA deletion detection assays.

### Testing the sensitivity of ND4^−^ template detection using model, deleted mtDNA substrates

**Figure 4:**
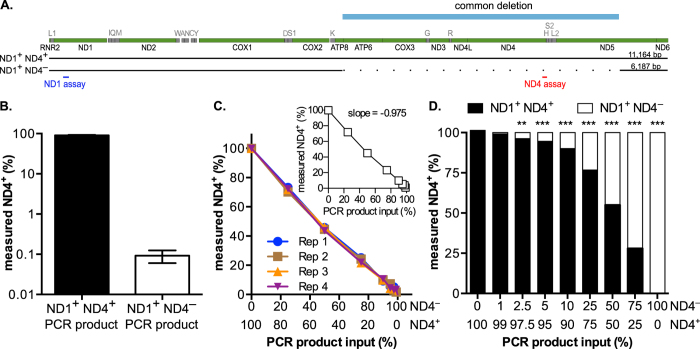
Sensitivity of ND4^−^ detection using model Δ mtDNA substrates. (**A**) Cartoon of mtDNA PCR products used in the qPCR analysis (ND1^+^ND4^+^ and ND1^+^ND4^−^). (**B**) Relative percentage ND4^+^ detection on ND4^+^ and ND4^−^ substrates (average +/− SEM of triplicate reactions). (**C**) ND4^+^ detection in four replicate experiments is linear at all tested mixtures of ND4^+^ and ND4^−^ PCR products. Inset: Average of the four replicate experiments. Slope was determined by linear regression analysis (r^2^ ≥ 0.99). (**D**) Comparison of input compared to measured values of ND4^+^ content (black bars) and ND4^−^ content (white bars). Significance was determined by one-way ANOVA with Dunnett’s posthoc comparison to 100% ND4^+^ (p-values: ** < 0.01; *** < 0.001).

To test the linearity of detection of the most common mtDNA deletion (Fig. 4), we next generated two PCR products, one with ND1 and ND4 intact (ND1^+^ND4^+^) and a second one harboring the so-called “common” deletion sequence lacking ND4 (ND1^+^ND4^−^) (Fig. 4A). Using these purified products, we observed a low false positive rate for ND4^+^content of 0.1% using the ND1^+^ND4^−^ template possibly due to basal probe hydrolysis during cycling (Fig. 4B). To assess the sensitivity of the qPCR assay in detecting mtDNA deletions on an optimal double stranded substrate, we mixed the ND1^+^ND4^+^and ND1^+^ND4^−^ templates in varying proportions. As shown by the overlapping lines, the measured ND4^+^ content was consistent between all four replicate experiments (Fig. 4C) and linear across the range tested (Fig. 4C, inset). More importantly, however, was that the limit of detection of ND4^−^ templates for the assay is ~2.5% of the total amount of mtDNA (Fig. 4D). By using our defined ND1 normalization parameters, this multiplex assay achieves strong correlation between the expected and observed ND4^−^ content yielding highly sensitive and precise mtDNA deletion (ND4^−^) detection.

### Quantitative PCR to determine the limit of detection of ND4-deleted mtDNA

Prior to testing cellular or patient DNA, it is important to note that PCR products often serve poorly as external calibrants for mtDNA ∆ content of cellular DNA. This is because the ∆C_q_ values of ND4 minus ND1 can be quite different to the ∆C_q_ values from a cellular template, as the amplification characteristics of these sequences differ. This result also highlights concern for the use of PCR products as a calibrant for mtDNA content.

**Figure 5:**
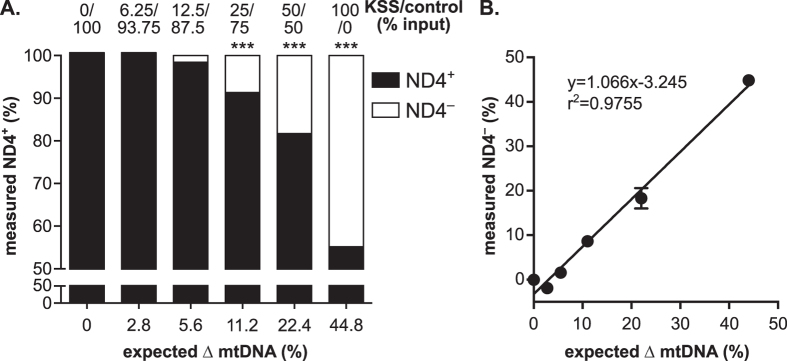
Titration of cellular DNA to determine the detection limits of ND4^−^ mtDNA deletions. (**A**) Titration of total DNA from control and KSS patients. Shown is the percent input of control and KSS patient mtDNA with expected ND4^−^ (∆ mtDNA) contribution indicated. Significance was determined by one-way ANOVA with Dunnett’s posthoc comparison to zero expected ∆ mtDNA sample on technical triplicates (p-values: *** < 0.001). (**B**) Comparison of ∆ mtDNA (ND4^−^) input to measured values of ND4^−^ content. Slope and r^2^ was determined by linear regression analysis for entire dataset.

Thus, to test the limits of detection in patient cell lines, we prepared total DNA from control human and “common deletion” KSS patient fibroblast cell lines, which have similar ND1 content per nuclear genome (Fig. 2F). Based on the ND1 content from Fig. 2, we mixed total DNA from control and KSS patient samples in varying ratios and used the ND4 and ND1 TAQMAN assays in qPCR to assess the limits of detecting ND4^−^ (∆ mtDNA) (Fig. 5). The KSS patient cell line carrying the common ∆ mtDNA, compared to the control fibroblasts (assumed 0% ∆ mtDNA), showed 44.8% ∆ mtDNA by this assay, which is in close agreement with published Southern blot estimates of 44% for this line^37^. By mixing the KSS and control DNA preparations (balanced for ND1 content), we found that the detection limit for decreased ND4^+^ content is ~10% of the total mtDNA content (Fig. 5A). The input contribution of each DNA preparation was used to calculate the expected mtDNA deletion load using the value 44.8% for 100% KSS samples, and 0% for 100% control sample. We observed a strong linear correlation with an r^2^ of 0.9755 for the whole range of data between expected and measured ND4^−^ content in this assay. The linear regression plot intercepts the X-axis at ~3% ∆ mtDNA template input (Fig. 5B). The association between expected and measured deletion load is noticeably diminished at low deletion mixture. We surmise that there are some differences in amplification characteristics of the two DNA templates because the idealized PCR templates used in Fig. 4 showed greater consistency across all deletion ranges. The analysis of low mtDNA deletion abundance highlights the limitations of using an external control reference.

### Digital PCR to determine the limit of detection of ND4-deleted mtDNA

**Figure 6:**
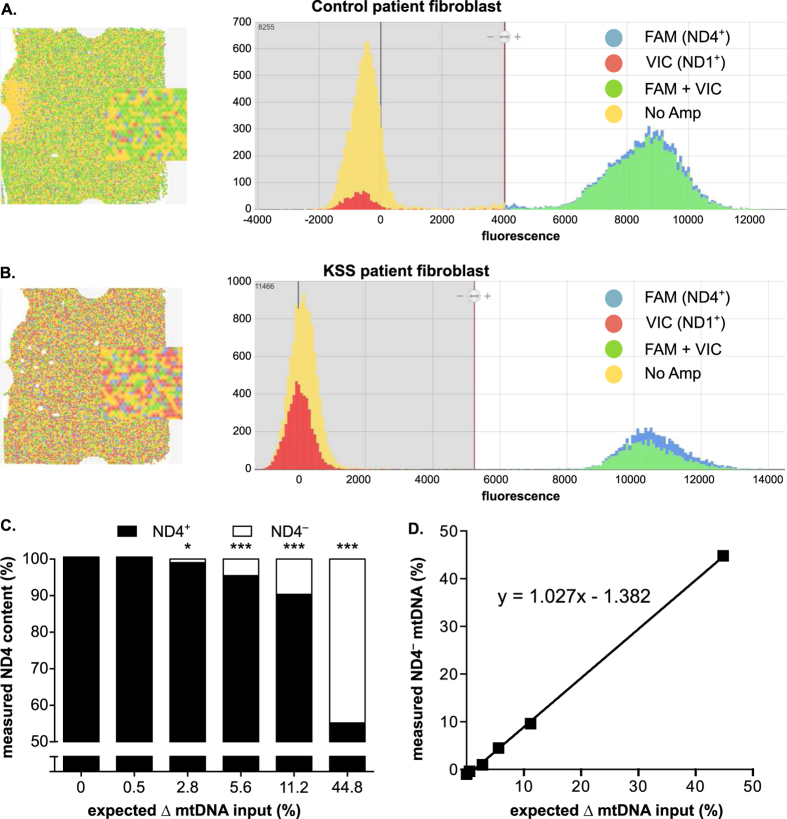
Digital PCR (dPCR) assessment of minimal mtDNA deletion loads. Representative chips and histograms diagram show the number and distribution of ND1 and ND4 positive wells in DNA isolated from (**A**) control patient fibroblast and (**B**) KSS patient fibroblast lines. (**C**) Total DNA from control and KSS fibroblast cells were mixed in varying proportions. Each mixture was assayed for ND4^+^ and ND1^+^ on triplicate chips and the average was used to calculate relative ND4 content. The ND4/ND1 ratios that were used to calculate the ∆ mtDNA are not normalized to the non-deleted sample. Significance among the technical triplicates was determined by one-way ANOVA with Dunnett’s posthoc comparison to zero expected ∆ mtDNA sample (p-values: * < 0.05; *** < 0.001). (**D**) Comparison of ∆ mtDNA (ND4^−^) input to measured values of ND4^−^ content. Slope was determined by linear regression analysis for entire dataset (r^2^ > 0.999).

To both eliminate the need for reference samples and improve the precision of the ND1 and ND4 assays, we performed digital PCR with those assays on the control and KSS patient fibroblast DNA (Fig. 6). The digital PCR approach involves the partitioning of dilution-limited DNA into ~20,000 wells of ~865 pL each prior to amplification (using V1 chips). Post-amplification fluorescence detection determines whether sample was loaded into a well (i.e. ROX-dye positive). Among those wells, scoring above signal threshold is made for the number of wells that are PCR positive for each assay versus wells with no amplification. It is necessary to have “no amplification” rates between ~20% to fit a Gaussian distribution, which is used to determine the number of ND4^+^ or ND1^+^ events on the chip. As would be expected from an endpoint assay, we do not observe differences in results between multiplex and singleplex reactions (Supplementary Fig. S1).

Using digital PCR, we observed several distinguishing features between the control and KSS patient fibroblast samples used in this analysis. First, representative chip images show that the KSS patient sample (Fig. 6B) contains greater ND1^+^ -only events, represented by the red dots on the chip and the corresponding red histogram, when compared with the control (Fig. 6A). Second, absolute quantity of ND4 (copies/μL) divided by that of ND1 in the patient and no-deletion control DNA yield a 44.8% ∆ mtDNA burden for the KSS cell line – identical to that detected by qPCR in Fig. 5. In dPCR, this value was achieved without the need to normalize to a control or external reference sample. In experiments with mixed proportions of control and KSS fibroblast total DNA, the limit of detection for ND4^−^ content was improved to 2.8% with digital PCR (Fig. 6C). The expected mtDNA deletion values were calculated based on the input contribution of each DNA, as was done in Fig. 5. Further linear regression analysis of the data shows that detection of mtDNA deletion load via dPCR is linear above 1% of the total calculated deletion load, with strong correlation (r^2^ > 0.999) between the measured and calculated ND4^−^ content (Fig. 6D). Because dPCR generates absolute copies/μL, rather than relative data in qPCR, normalization to control has minimal effects (Supplementary Table S1). With dPCR, the overall assay variability among technical replicates (n = 3 chips per mixture) is low without normalization (Supplementary Table S1).

### Comparison of qPCR to dPCR on patient biopsy samples

To test the potential clinical benefit of dPCR in detecting ND4, we analyzed total cellular DNA isolated from frozen vastus lateralis muscle biopsies from control, POLG1 mutation patients, and CPEO individuals. The POLG1 patient population is affected by mitochondrial respiratory chain dysfunction, occasionally with mtDNA depletion or mtDNA deletions^38^. POLG1 mutations identified in this cohort are reported in Supplementary Table S2. CPEO can develop at any age, and patients generally present with PEO and proximal muscle weakness, with varying incidence of ptosis, hearing loss, muscle weakness, and dysphagia. Numerous mtDNA abnormalities can manifest as CPEO. The m.3243A > G mutation, also responsible for mitochondrial encephalomyopathy, lactic acidosis, and stroke-like episodes or MELAS, has been shown to associate with CPEO^39^, as have the presence of a single mtDNA deletion (such as the “common deletion”) or multiple mtDNA deletions (i.e. various mixed forms)^40^. The CPEO patient cohort presented here were long-range PCR positive for mtDNA deletions, but lacked any obvious familial disease inheritance and were all adult onset, which is commonly found in sporadic cases (Supplementary Table S3).

**Figure 7:**
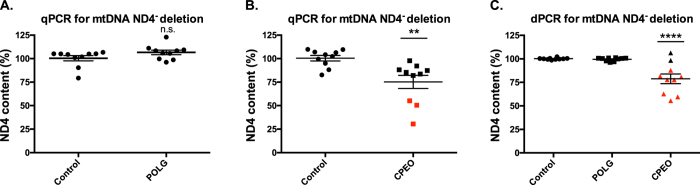
Comparison of qPCR and dPCR analyses of control, POLG1, and CPEO patient groups. (**A**) qPCR for mtDNA ND4- deletions in control and POLG1 patient groups. (**B**) qPCR for mtDNA ND4- deletions in control and CPEO patients. (**C**) dPCR for mtDNA ND4- deletions in control, POLG1, and CPEO patient groups. Each sample group was comprised of ten patients. Individual samples were analyzed in technical triplicates and averaged. Significance in Panels A and B were determined by Students t-test, whereas Panel C was determined by one-way ANOVA with Dunnett’s posthoc comparison (p-values: ** < 0.01, **** < 0.0001). Data points in red differed from 100% ND4^+^ mtDNA by greater than two-fold the standard deviation of the control group for each technique.

We first characterized the control, POLG1, and CPEO patient samples for mtDNA abundance and ND4^−^ deletion content using qPCR. The mtDNA content was measured using B2M and ND1 probe sets in multiplex reactions (Supplementary Fig. S2). Although a trend toward mtDNA depletion was observed in the POLG1 sample group, it did not achieve statistical significance. Consistent with previous reports^41^, no mtDNA depletion was observed in the CPEO patient samples. After normalizing for ND1 content among the samples, we next measured the ND4^−^ deletion frequency using ND4 and ND1 probe sets in multiplex reactions, first on control vs POLG1 samples, then on control vs CPEO samples (Fig. 7). We found no statistical support for ND4^−^ deletions in the POLG1 patient cohort (Fig. 7A), which do not frequently show mtDNA deletions^38^. In contrast, the CPEO patient cohort showed significant ND4-depletion by qPCR (p < 0.01) (Fig. 7B).

We next characterized each sample by digital PCR and calculated the ND4^−^ deletion load. In general, we observed less variation in deletion burden within each sample population using dPCR compared to qPCR. The improved accuracy of deletion detection and precision of each group yielded greater statistical support for differences between CPEO patients and control patients (p < 0.0001) (Fig. 7C). The control group showed an improvement in eliminating “false positive” mtDNA deletions using dPCR compared to qPCR. All patient dPCR results are summarized in Supplementary Table S4.

Importantly, when considering the individual patient diagnosis, the dPCR method far outperformed the qPCR analysis. Using the criteria for molecular diagnosis that ND4-depletion must exceed two-times the standard deviation of the control group (>18.45 deletion), the qPCR assay would identify three patients (colored red in Fig. 7B), while missing several patients that had been shown to contain mtDNA deletions through long extension PCR (data not shown). Using the same criteria for the dPCR assay (>1.935 deletion), eight patient samples would be indicated for elevated ND4-deletion, including all of the long PCR positive samples. Overall, these criteria are in agreement with our estimated limits of detection in Fig. 5. None of the POLG1 patient samples were identified as carrying a significant ND4-deletion burden, suggesting a limited occurrence of false positives with the dPCR method.

## Discussion

The existing methods to detect mtDNA deletions, such as Southern blot, long amplification PCR, and qPCR have variable sensitivity and are limited in their capacity to quantify low abundance mtDNA deletions. The purpose of our study was to precisely define the assay parameters to maximize the deletion detection limit using qPCR and dPCR methodologies. Specifically, we quantified ND4 content because this is the most frequently deleted genomic region observed in mtDNA deletions^9^,^10^. We utilized PCR-amplified substrates to define these parameters on idealized templates; however, cell lines showed decreased sensitivity for mtDNA deletion detection potentially due to limitations in amplification efficiency on complex templates^42^.

To overcome this limitation in patient samples, we introduced a digital PCR method that improves precision to reduce error and increases sensitivity for ND4 and ND1 TAQMAN assays used in relative qPCR. The precision of dPCR in detecting copy number variation arises from the principle of the technique, which measures thousands of single molecule PCR events. Therefore, one requirement is that a sufficient number of PCR events must be measured on the chip to prevent small variations from contributing to error but without saturating chip occupancy to allow fitting the data to a Poisson distribution. To our knowledge, dPCR has not been tested to directly measure low abundance mtDNA deletions^32^. From patient samples, we are able to conclude that dPCR is strongly preferable to qPCR when deletion loads are moderate (5–15%) and that qPCR is adequate for samples containing higher ND4 deletions (<25%).

The application of dPCR, however, does not displace the benefits of next generation sequencing, especially when molecular diagnosis of the causative nuclear gene is desired. Whole exome sequencing of mtDNA requires thousands of reads to estimate deletion load, which decreases the quality of nuclear genome coverage. To overcome this challenge, many approaches include a mtDNA amplification step to reduce the detection of nuclear mitochondrial DNA sequences (NUMTs) for a dedicated mtDNA sequencing analysis. This increases the cost and time for analysis and bioinformatics, as well as generates false negative readouts depending on the position of mtDNA deletion breakpoints and the primers selected. The NGS approach for estimating mtDNA deletions will yield specific breakpoints when a deletion is adequately enriched. In contrast, the dPCR methods described here specifically measure the frequency of ND4-deletion normalized to ND1 levels. This shows a disadvantage of dPCR with these probes, which will overlook deletions lying outside of the ND4 region. Overall, the sensitivity of dPCR and NGS for deletion detection are similar^29^.

dPCR technology allows the detection of less common mtDNA deletions, which is not limited to skeletal muscle, without laser capture enrichment^20^,^21^,^22^, restriction enrichment^33^, or deletion biased PCR^23^,^24^,^25^,^26^. dPCR can be completed and analyzed before the long-amplification product is purified for NGS library generation, allowing early assessment of mtDNA stability. It is expected that applying additional probes, increasing the number of fluorophor channels, and other technological improvements will allow rapid and simple assessment of multiple types of mtDNA deletions directly from biopsy material. It is also expected that this technology will allow the detection of small but significant alterations in mtDNA deletion formation in other indications.

## Methods

### Cellular DNA preparation

Total cellular DNA was prepared as published^42^. In brief, HeLa cells (ATCC CCL-2), F31 and ∆ fibroblast cell lines^37^ were cultured in DMEM (4 mM L-glutamine, sodium pyruvate, 4.5 g/L glucose; GE, Logan, UT) plus 5% FBS + 5% FCS with pen-strep (1%). Sub-confluent, cells were collected by trypsinization, neutralized with media, and pelleted. Cells were resuspended in PBS, transferred to 1.6 ml Eppendorf tubes and pelleted. After aspiration, cells were snap frozen in dry ice and stored at −80 °C. Cells were rapidly thawed and resuspended in Proteinase K buffer (100 mM Tris-HCl pH 8.5, 5 mM EDTA, 0.2% SDS, 200 mM NaCl) and digested overnight. Lysate was adjusted to 5 M NaCl and rocked for 5 min prior to clarification. Supernatant was transferred to clean tubes and nucleic acids precipitated with ethanol. Pellet was air dried overnight and gently resuspended at 37 °C in TE buffer containing RNase A.

### Traditional PCR Primers

All primers were from IDT (Coralville, IA):

Primers used to generate the ND1-ND4 PCR product:

 -hND1 fwd; 5′-ACC CAC ACC CAC CCA AGA AC-3′

 -hND4 rev; 5′-AGG GTG GTT ATA GTA GTG TGC ATG G-3′

Primers used to generate the ND1^+^ND4^+^ and ND1^+^ND4^−^ PCR products:

 -hND1 fwd; 5′-ACC CAC ACC CAC CCA AGA AC-3′

 -hND6 rev; 5′-GTG CTG TGG GTG AAA GAG TA-3′

### Amplification parameters to generate purified double-stranded templates for calibration

To generate reference PCR products, LongAmp 2X Mastermix (NEB, Ipswich, MA) was used in a 25 μL final reaction volume and PCR amplification was performed in a ProFlex thermal cycler (Applied Biosystems, Waltham, MA) with the following PCR amplification profile: 94 °C for 30 sec; 30 cycles of 94 °C for 20 sec, 55 °C for 20 sec, and 65 °C for 8 min; and 65 °C for 10 min. Template concentration was 1 ng/reaction while primer concentration was at 4 μM. The double-stranded linear PCR product containing the ND1 and ND4 targets used in Fig. 3 was generated from total HeLa DNA using the hND1f and hND4r primers. The ND1^+^ND4^+^ and ND1^+^ND4^−^ PCR products used in Fig. 4 were generated by PCR using total DNA isolated from HeLa (ND1^+^ND4^−^) and KSS patient (carrying the common deletion or ND1^+^ND4^−^ sequence) cells and hND1f and hND6r primers. For the ND1^+^ND4^−^ PCR product, the template DNA was restricted with the Eco105I* enzyme (Thermo) to prevent WT mtDNA amplification. All products were resolved on 0.5% 1X TBE agarose, gel purified (Qiagen, Germantown, MD), and quantitated by OD260 (Nanodrop 1000, Thermo).

### Primer-probe assays

A primer: probe ratio of 3:1 was used for all assays, except for the primer-limited form (ND1pl), which was used at 1:1 for mtDNA quantitation. All primers and probes were from IDT (Coralville, Iowa).

ND1 assay

Probe: 5′-HEX/CCATCACCC/ZEN/TCTACATCACCGCCC/3IABkFQ/-3′

Primer 1: 5′-GAGCGATGGTGAGAGCTAAGGT-3′

Primer 2: 5′-CCCTAAAACCCGCCACATCT-3′

ND4 assay

Probe: 5′-FAM/CCGACATCA/ZEN/TTACCGGGTTTTCCTCTTG/3IABkFQ/-3′

Primer 1: 5′-ACAATCTGATGTTTTGGTTAAACTATATTT-3′

Primer 2: 5′-CCATTCTCCTCCTATCCCTCAAC-3′

B2M assay

Probe: 5′-FAM/ATGTGTCTG/ZEN/GGTTTCATCCATCCGACA/3IABkFQ/-3′

Primer 1: 5′-TCTCTCTCCATTCTTCAGTAAGTCAACT-3′

Primer 2: 5′-CCAGCAGAGAATGGAAAGTCAA-3′.

### Quantitative PCR method and parameters

All quantitative PCR (qPCR) assays used TaqMan Universal Master Mix (Applied Biosystems, Waltham, MA) and 5 μM primer/probes in 15 μL reactions performed on a StepOnePlus thermo cycler (Applied Biosystems) at the following standard thermal parameters: 50 °C for 2 min, 95 °C for 10 min and 40 cycles of 95 °C for 15 sec and 60 °C for 1 min. Multiplex or singleplex assessment of relative mtDNA copy content was performed on total DNA isolated using ND1pl and B2M primer/probes and calculated using the ∆∆C_q_ method^24^. Serial dilutions were performed to assess linearity of assay conditions. Multiplex reactions were used to assay relative ND4 and ND1 content^24^,^43^. Calculation of deletion load (ND4^−^ content) was as described^24^,^43^, where ND1 C_q_ values were subtracted from ND4 C_q_ values for each replicate and then normalized to the control fibroblast samples using the ∆∆C_q_ method.

### Digital PCR method and parameters

Total DNA from control and KSS fibroblast cell lines were used as templates for digital PCR (dPCR) analysis using multiplex ND4 and ND1 primer probes on the QuantStudio 3D Digital PCR System and with supplied reagents (Applied Biosystems, Waltham, MA). In titration experiments, relative mtDNA inputs were normalized by the ND1 assay in qPCR as determined above. The dPCR was prepared according to the manufacturer’s protocol and loaded onto the individual QuantStudio v1 dPCR chips using the QuantStudio 3D Digital PCR Chip Loader. Chip PCR amplification was performed in a Proflex 2× flat block thermal cycler (Applied Biosystems, Waltham, MA) using standard conditions: 96 °C for 10 min; 39 cycles of 60 °C for 2 min and 98 °C for 30 sec; and 60 °C for 2 min. Chips were then read on the QuantStudio 3D instrument to obtain the number of wells positive for the VIC/HEX and FAM channels, the number of wells without DNA (ROX positive, VIC and FAM negative) and empty wells (ROX negative). Data analysis and chip quality were assessed using the QuantStudio 3d Analysis Suite. All chips were between 25–75% empty well ensuring suitability for quantitation. The number of ND4^+^ wells was divided by the total ND1^+^ wells to calculate the fraction of normal (WT) mtDNA content. The ND4^−^ content is one minus that value. All dilutions were performed in at least triplicate to determine error.

### Statistical Analyses

Statistical analysis was performed using GraphPad Prism software (GraphPad, La Jolla, CA). The Student two tailed, unpaired T-test was used to determine the significance of differences between two groups, whereas one-way ANOVA with Dunnett’s post-hoc analysis was used for series of samples compared to control. Linear regression analyses were performed in order to obtain the slope values and correlation coefficients (r^2^). P-values of <0.05 were accepted as a significant difference. Standard error of the mean is shown for all experiments but not all bars are visible.

## Additional Information

**How to cite this article**: Belmonte, F. R. *et al.* Digital PCR methods improve detection sensitivity and measurement precision of low abundance mtDNA deletions. *Sci. Rep.* **6**, 25186; doi: 10.1038/srep25186 (2016).

## Supplementary Material

Supplementary Information

## Figures and Tables

**Figure 1:**
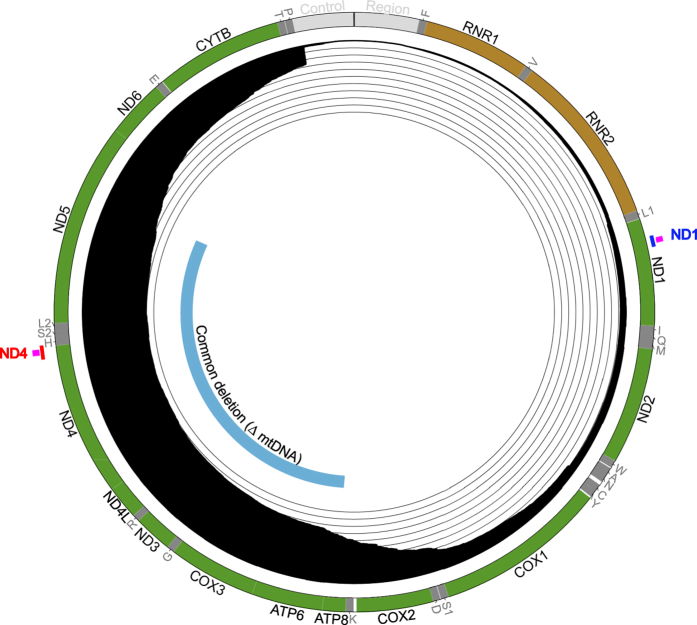
Diagram of human mtDNA genome content, deletions and probes used in this study. Human mtDNA (nc_012920) contains 16,569 base pairs numbered from the top of the Circos diagram and proceeding clockwise. Protein coding genes are colored green and labeled in black; Ribosomal RNAs are labeled in black and colored brown; transfer RNAs are labeled and colored dark gray; and, the replication/transcription control region is labeled and colored in light gray. The frequency of deletions for each mtDNA position is presented inside of the mtDNA ideogram, with each ring representing a 0.1 frequency increment (~80 deletions). The innermost track depicts the location of the common deletion (8483–13459), which is labeled with black text and colored blue. The inside of the mtDNA ideogram shows the ND4 amplicon used in this study, labeled and colored in red, and the ND1 amplicon, labeled and colored in blue. Probes used for these assays are indicated in pink at their respective locations.
