# Supplementary material for: Digital PCR methods improve detection sensitivity and measurement precision of low abundance mtDNA deletions
Source: Sci Rep. 2016 Apr 28;6:25186. doi: 10.1038/srep25186 (PMC4848546; doi:10.1038/srep25186)
Supplement: Supplementary Information [file srep25186-s1.pdf]

Digital PCR methods improve detection sensitivity and measurement precision of low abundance mtDNA deletions

Frances R. Belmonte[1], James L. Martin[1], Kristin Frescura[2], Joana Damas[3], Filipe Pereira[4], Mark A. Tarnopolsky[2], and Brett A. Kaufman[1*]

[1]University of Pittsburgh School of Medicine, Division of Cardiology, Center for Metabolism and Mitochondrial Medicine and Vascular Medicine Institute, Pittsburgh, PA, United States, 15261. [2]Departments of Pediatrics and Medicine, McMaster University Medical Center, Hamilton, ON, Canada, L8N 3Z5. [3]Department of Comparative Biomedical Sciences, Royal Veterinary College, London, UK. [4]Interdisciplinary Centre of Marine and Environmental Research (CIIMAR), University of Porto, Porto, Portugal.

*Communicating author: bkauf@pitt.edu

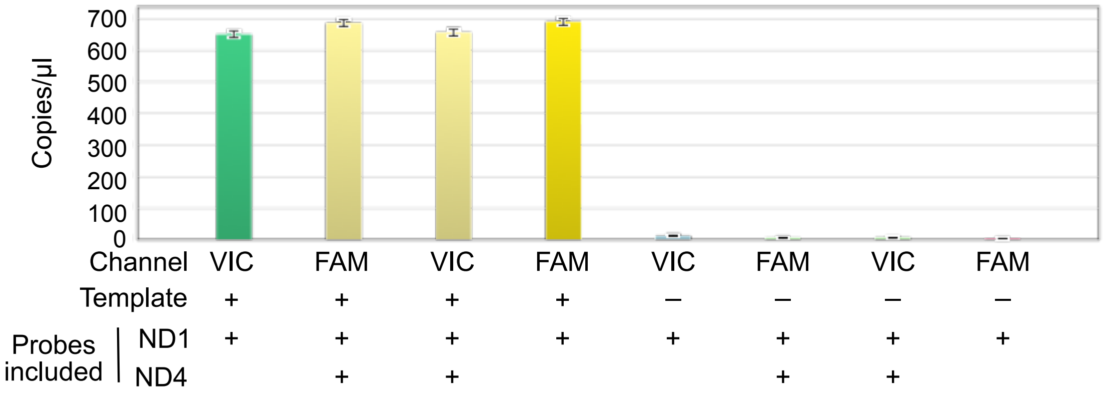

Supplementary Figure S1. Comparison of dPCR results between singleplex and multiplex reactions on HeLa DNA. Number of copies of each target was unaffected by the presence of the other probe. ND1 is detected in the VIC channel, ND4 is detected in the FAM channel.

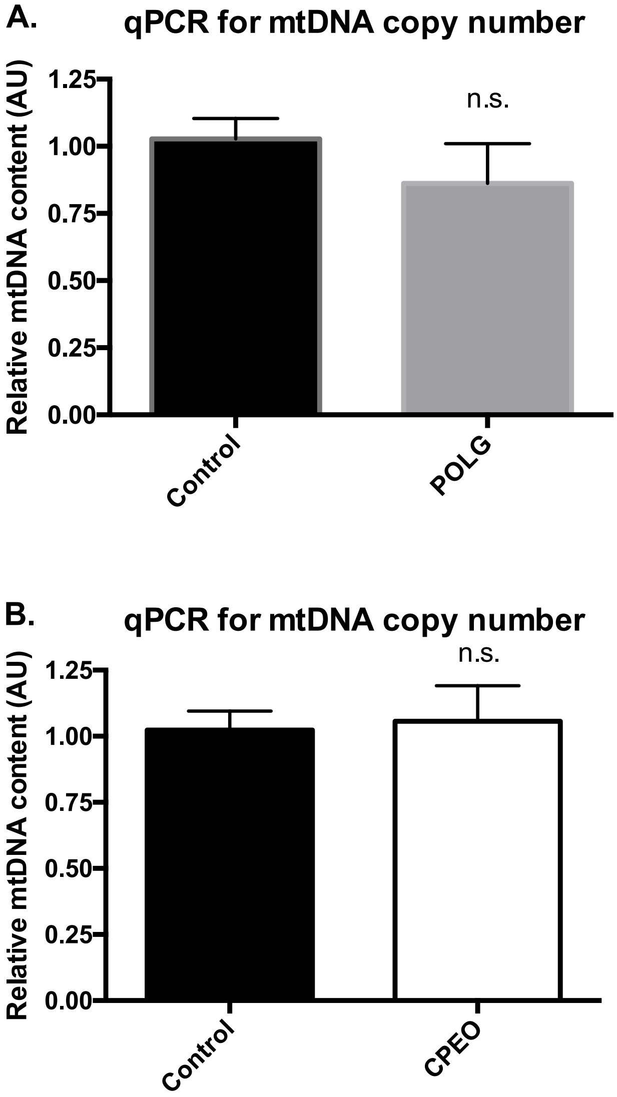

Supplementary Figure S2. Additional features of control, POLG, and CPEO patient mtDNA. Relative mtDNA content from control, POLG, and CPEO patients. A) Relative mtDNA abundance of POLG patients relative to control samples. B) Relative mtDNA abundance of CPEO patients relative to control samples. (n=10 per group)

Sample mixture	$ND4^+$ / $ND1^+$ (copies/µL)	$ND4^+$ (%)	SD	Normalized $ND4^+$ (%)	SD	$ND4^-$ (%)	p-value
0 control: 100 KSS	453.7/ 823.1	55.16	1.101	54.63	1.093	45.37	***
75 control: 25 KSS	822.9/ 911.5	90.22	0.632	89.41	0.635	10.59	***
87.5 control: 12.5 KSS	915.1/ 959.0	95.43	0.864	94.57	0.857	5.432	***
93.75 control: 6.25 KSS	938.3/ 948.2	98.95	0.469	98.06	0.463	1.941	*
99 control: 1 KSS	962.5/ 959.9	100.3	0.454	99.38	0.454	0.6166	ns
100 control: 0 KSS	849.5/ 842.5	100.9	0.963	100.0	0.964	0	N/A

Supplementary Table S1. Absolute quantities of ND4/ND1 measured by digital PCR. Sample mixtures were assayed on triplicate chips. Normalized ND4/ND1 ratios were based on the control sample containing zero deletions. Significance among the technical triplicates was determined by one-way ANOVA with Dunnett's posthoc comparison to pure control DNA sample without normalization (p-values: *<0.01; ***<0.001).

POLG1	Diagnosis	RQ	ND4+ mtDNA (%)	Gene	Result	Mutation Type	Family Studies
A	Mitochondrial cytopathy and HSP Type 7	0.63	101	POLG1	c.32G>A	Pathogenic	Not Done
B	CPEO	1.72	100.43	POLG1	Het c.2496T>G	Pathogenic	Segregates
C	Complex mitochondrial cytopathy	0.44	96.21	POLG1	c.A2852C:p.Y951S	Pathogenic	Likely de novo
D	Non-mitochondrial disorder	1.32	99.89	POLG1	c.1550G>T: p.G517V	Variant of Unknown Significance	Unassociated
E	Mitochondrial cytopathy	0.38	100.33	POLG1	W748S; G48S	Pathogenic	Not Done
F	SCA26	1.37	100.8	POLG1	c.2243G>C	Likely Benign (or recessive mutation)	Not Done
G	CPEO	0.42	96.92	POLG1	Het c.2496T>G	Pathogenic	Segregates
H	CPEO	0.91	97.8	POLG1	Het c.2496T>G	Pathogenic	Segregates
I	Mitochondrial cytopathy	0.87	101.19	POLG1	p.Ser1080Thr	Possibly pathogenic	Unclear
J	Secondary mitochondrial dysfunction	0.57	100.65	POLG1	Gln1236His	Polymorphism	Not Done

Supplementary Table S2. POLG patient mutation information with familial assocation. Relative mtDNA quantity (RQ) and ND4+ mtDNA are from this study.

CPEO	ND4+ mtDNA (%)	Sex	Age at biopsy	Age of Onset	Family History	Ptosis	Opthalmoplegia	Myopathy	Muscle Weakness	Muscle Atrophy	Fatigue	Exercise Intolerance	Ataxia	Constipation	Dysphagia	Hearing Loss	Muscle Biopsy	Other
A	98.3	F	66	50s	No	1	1		1 - Mild Proximal Weakness	0	Yes	0	0	0	0	High Frequency Hearing Loss	COX deficiency, Type II Fibre Atrophy	COPD, Type 2 Diabetes, Migraines, Depression
B	87.75	F	70	45	No	very mild - 0/1	1	1 - Promixal	1 - Mild Proximal	0	No	0	1 - frequent falls	0	0	Sensorineural Hearing Loss	--	Depression, Osteoporosis, Blepharoplasty
C	55.46	M	32	Unknown	No	1	1	1 - Proximal	1 - Mild Proximal	0	No	0	0	0	0	No	--	--
D	81.44	F	56	44	No	1	1	1	1 - Mild Proximal Weakness	0	No	0	0	0	1	High Frequency Hearing Loss	COX deficiency, Electron Dense Mitochondria, Lipid Myopathy	Sporadic Diplopia, Depression, Osteoporosis, Nasal Dysarthria, Chronic Tinnitus
E	59.55	F	48	40s	No	1	1	1	1 - Mild Proximal Weakness	0	No	0	0	0	0	No	--	--
F	62.7	F	46	20	Maternal hearing impairment	1	1	1	1 - Mild Proximal Weakness	0	No	0	0	1	1	High Frequency Hearing Loss	COX deficiency, Minimal denervation, Type II fibre atrophy	Depression, Anxiety, Headaches
G	80.7	M	63	60s	No	0	1	0	0	1 - Generalized	No	0	0	0	0	High Frequency Hearing Loss	Focal COX Deficiency, Denervation without Reinnervation, Microangiopathy - Moderate	Nasal Dysarthria, Diplopia, Severe Osteoarthritis
H	77.69	M	53	40s	No CPEO - suicide and depression	1	1	0	0	0	No	0	0	0	1	Hearing Loss	COX Deficiency, Red Ragged Fibres, Microangiopathy (Severe), Mild Denervation without Reinnervation	Depression, Headaches, Diplopia, Dysesthesias in the Feet, Smoker
I	77.73	F	71	67	No	1	1	1	1 - Mild Proximal Weakness Upper Extremities	0	No	0	0	0	0	Hearing Loss	COX deficiency, Slight Microangiopathy, Slight Type 2 Muscle Fibre Preponderance	Exotropia, Diplopia, Diabetes, Decreased Visual Acuity
J	106.34	F	53	40s	No	1	1	1 - Proximal	1 - Proximal Upper and Lower	0	Yes	1	0	1	0	Hearing Loss	Partial COX Deficiency, Focal Fibre Atrophy (Non-Specific)	Tinnitus, Aural Fullness, Hypothyroidism, Diplopia, Cluster Headaches, IgA Nephropathy, Meniere's Disease

Supplementary Table S3. CPEO patient clinical data showing age of onset and absence of familial history of mitochondrial disease. All patients were long-range PCR positive for mtDNA deletions.

Group	Patient	ND4 / ND1 (copies/μL)	$ND4^{+}$ mtDNA (%)
Control	A	1290.50 / 1289.80	100.05
Control	B	1039.70 / 1041.90	99.79
Control	C	642.03 / 639.53	100.39
Control	D	1269.80 / 1262.40	100.59
Control	E	2642.00 / 2630.80	100.43
Control	F	839.80 / 844.85	99.41
Control	G	1003.90 / 981.55	102.28
Control	H	982.57/996.33	98.62
Control	I	1615.80 / 1614.30	100.09
Control	J	544.72 / 547.94	99.41
**Group**	**Patient**	**ND4 / ND1 (copies/μL)**	**$ND4^{+}$ mtDNA (%)**
POLG	A	648.74 / 642.32	101.00
POLG	B	707.51 / 704.45	100.43
POLG	C	938.09 / 975.00	96.21
POLG	D	1662.60 / 1664.40	99.89
POLG	E	466.85 / 465.31	100.33
POLG	F	584.86 / 580.23	100.80
POLG	G	988.84 / 1020.30	96.92
POLG	H	570.84 / 583.71	97.80
POLG	I	924.76 / 913.91	101.19
POLG	J	794.55 / 789.43	100.65
**Group**	**Patient**	**ND4 / ND1 (copies/μL)**	**$ND4^{+}$ mtDNA (%)**
CPEO	A	479.68 / 487.98	98.30
CPEO	B	689.02 / 785.17	87.75
CPEO	C	320.64 / 578.13	55.46
CPEO	D	517.03 / 634.87	81.44
CPEO	E	290.06 / 487.06	59.55
CPEO	F	249.44 / 397.81	62.70
CPEO	G	630.25 / 780.94	80.70
CPEO	H	421.74 / 542.82	77.69
CPEO	I	377.69 / 485.88	77.73
CPEO	J	591.75 / 556.45	106.34

Supplementary Table S4. Calculations for ND4-deletion in control, POLG, and CPEO patient groups. Red values are patients showing more than two-times the SD of control from 100% $ND4^{+}$ by dPCR and were plotted in Figure 7.
